# The Application of Nitric Oxide for Ocular Hypertension Treatment

**DOI:** 10.3390/molecules26237306

**Published:** 2021-12-01

**Authors:** Binze Han, Maomao Song, Liping Li, Xinghuai Sun, Yuan Lei

**Affiliations:** Department of Ophthalmology & Visual Science, Eye & ENT Hospital, Shanghai Medical College, Fudan University, Shanghai 200031, China; Hanbz2014@126.com (B.H.); 17111260011@fudan.edu.cn (M.S.); 21111260010@fudan.edu.cn (L.L.)

**Keywords:** glaucoma, conventional outflow system, intraocular pressure, NO donors

## Abstract

Despite of various therapeutic methods for treating ocular hypertension and glaucoma, it still remains the leading cause of irreversible blindness. Intraocular pressure (IOP) lowering is the most effective way to slow disease progression and prevent blindness. Among the ocular hypotensive drugs currently in use, only a couple act on the conventional outflow system, which is the main pathway for aqueous humor outflow and the major lesion site resulting in ocular hypertension. Nitric oxide (NO) is a commendable new class of glaucoma drugs that acts on the conventional outflow pathway. An increasing number of nitric oxide donors have been developed for glaucoma and ocular hypertension treatment. Here, we will review how NO lowers IOP and the types of nitric oxide donors that have been developed. And a brief analysis of the advantages and challenges associated with the application will be made. The literature used in this review is based on Pubmed database search using ‘nitric oxide’ and ‘glaucoma’ as key words.

## 1. Introduction

Glaucoma, one of the leading causes of irreversible blindness worldwide, refers to a group of diseases characterized by damage of the optic nerve and visual loss. Primary open angle glaucoma (POAG) is estimated to affect 111.8 million people by 2040 [1]. Intraocular pressure (IOP) elevation is a strong risk factor of POAG [2,3]. Therefore, research has been focused on better understanding the mechanism which is responsible for homeostatic regulation of aqueous humor outflow [4,5].

Since nitric oxide (NO) was discovered as a small gas signaling molecule mediating endothelial cell relaxation in 1987, it has been studied as a method of intervention for various diseases [6,7]. The mechanism by which NO lowers IOP includes the reduction of aqueous humor production [6,7], the increase of Schlemm’s canal (SC) endothelial cell permeability and the relaxation of trabecular meshwork (TM) cells [8,9]. The latter two effects can be boiled down to the increase of conventional outflow facility. The elucidation of these mechanisms provides the theoretical basis for the use of NO donors in the treatment of ocular hypertension.

A large amount of NO donors including conventional standalone NO donors, combination NO donors and NO donating nanomedicine have been proven effective in IOP lowering [5,10,11,12,13,14,15,16,17,18,19,20,21]. Combination therapies can have synergistic effect on IOP lowering, such as NO-donating prostaglandins, NO donating β-blockers, NO donating carbonic anhydrase inhibitor and the dual NO donor deliver system [22]. NO donor loaded nanoparticles improved the therapeutic effect and action duration of the conventional NO donor [20,21,22]. β-gal-NONOate aims to precisely deliver NO in a conventional outflow system [23].

In this review, the mechanism of NO in regulating IOP and the application of NO donor for the treatment of glaucoma and ocular hypertension will be introduced.

## 2. IOP Regulation in Physiological and Pathological Conditions

To understand how NO donors reduce IOP, it is important to know the mechanism of IOP regulation. Increased resistance to outflow through the TM and SC in the conventional outflow pathway leads to elevated IOP, which may distend the optic nerve head and damage RGC axons at the level of the lamina cribrosa [4,24]. This eventually leads to vision loss and blindness. IOP, which is determined by equilibrium in aqueous humor production and outflow through the conventional pathway and non-conventional system, is the only quantifiable risk factor for glaucoma [4,24]. The conventional outflow system consists concretely of the TM, the endothelial of SC, the juxtacanalicular connective tissue, the aqueous veins and the collecting channels. It provides pressure-dependent dynamic aqueous humor outflow [25,26]. The unconventional uveoscleral pathway is denominated a pressure-independent pathway. In physiological conditions, the aqueous formation and outflow is under homeostatic regulations [26]. However, if malfunction occurs in the conventional outflow system, the IOP elevates.

Dysfunction of conventional outflow system can be caused by multiple factors, including oxidative stress, cell autophagy, TM stiffness and genetic and environmental factors [3,26,27,28,29,30,31,32]. Evidence from previous research depicted a close correlation between human trabecular meshwork (HTM) damage by oxidative damage and elevated IOP in POAG patients [33]. Based on our data from angular aqueous plexus endothelial cells (AAP), which were isolated from porcine eyes and are equivalent to SC endothelial cells in human [34], chronic oxidative stress impacted on AAP cell barrier function by upregulating cytoskeleton and adhesion proteins [31]. Hyperoxic conditions were reported to contribute lysosomal activity impairment in the porcine TM cells, along with increased autophagic vacuole content [35]. Recently, autophagy in the conventional outflow system was shown to affect IOP [29,36]. The up-regulation of autophagosome-associated LC3 and p62, down-regulation of LAMP1 may have caused IOP elevation in DBA/2J mice through autophagic flux diminishment and the failure of the formation of autolysosomes in TM cells [36]. Increased stiffness of the TM may lead to increased conventional aqueous humor outflow resistance and IOP elevation [27,37,38,39]. TM stiffness depends on the interaction between the ECM and the resident TM cells [27,40]. Several factors, including senescence, TGF-β2, cytoskeletal disrupting agents, ROCK inhibitors, ocular corticosteroids and NO had been identified to affect TM stiffness [27,40,41,42,43].

## 3. NO and IOP Regulation

NO is produced by three isoforms of NO synthase namely nNOS, iNOS and eNOS by the conversion of l-arginine and molecular oxygen to l-citrulline. This process require several cofactors such as reduced nicotinamide-adenine-dinucleotide phosphate (NADPH), flavin adenine dinucleotide (FAD), flavin mononucleotide (FMN), and (6*R*-)5,6,7,8-tetrahydrobiopterin (BH_4_) [44,45,46,47]. In humans, eNOS expression was found in SC endothelial cells, ciliary body endothelium, TM and the ciliary muscle within the uveoscleral pathway [44,48,49,50]. nNOS is expression ciliary non-pigmented epithelium in human [51] and ciliary process epithelium in rat [52,53]. iNOS is expressed in the retina, retinal pigmented epithelium and outflow tissues [54,55]. NO bioavailability was connected with intake of nitrate salts [56].

Regulated by the increase of calcium-induced complex of calcium/calmodulin, nNOS and eNOS usually synthesized only a small quantity of NO and then played a role via NO/sGC/cGMP pathway or via S-nitrosylation modification for proteins [12,13,14]. The former pathway was closely related to IOP lowering; the latter may result in inflammation, apoptosis and cytotoxicity [44,57,58]. iNOS-induced NO production will also be accompanied by an inflammatory state and cause a large amount of continuous release of nitric oxide in a calcium-independent way, which in turn exacerbates the inflammation through the formation of ONOO- (peroxynitrite) [59,60,61]. iNOS activation and nitrotyrosine formation correlated with visual loss in in glaucoma patients [54,62].

**Figure 1:**
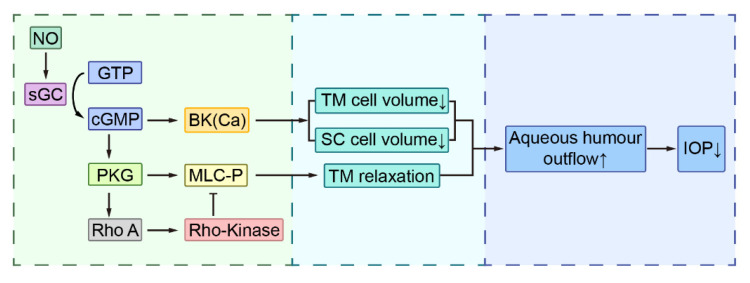
The mechanism of nitric oxide lowering intraocular pressure. TM, Trabecular Meshwork; SC, Schlemm’s canal; sGC, soluble guanylyl cyclase; IOP, intraocular pressure; PKG, protein kinase G; MLCP, myosin light chain phosphatase.

NO relaxes TM cells and makes SC cells more permeable [44,63,64,65,66,67,68,69,70]. The mechanism by which NO regulates IOP is illustrated in Figure 1. The IOP lowering function of NO is primarily mediated by the NO-cGMP pathway [71,72,73,74]. Experiment in rabbits has shown that both NO donors and the second messenger derivative (8-Br-cGMP) increased aqueous humor outflow facility dramatically [74]. An increased amount of evidence indicated the indispensable role of enzyme soluble guanylyl cyclase (sGC) in the process of NO-induced IOP lowering [75,76]. In GC-1 knockout mice, NO had little effect on IOP. GC-1 knockout and sGCα1 knockout mice showed impaired conventional outflow, sGC stimulator IWP-953 increased outflow facility [75,76,77].

TM and SC cell volumes were negatively correlated with outflow facility [64,68,78]. A study, performed in human SC cells, showed increased cell volume after L-NAME (NOS inhibitor) exposure. In contrast, NO donors DETA-NO and sodium nitroprusside (SNP) decreased cell volume in TM and SC cells [68,78]. Furthermore, in both TM and SC cell, BKCa channel activation was involved in NO-induced cell volume decrease and facility increase, which could be prevented by sGC inhibitor and IBTX, a BKCa channel inhibitor [68,78].

## 4. Nitric Oxide-Donating Drugs for IOP Lowering

With the invention and application of multiple NO-donating drugs, great progress has been made for NO donors in ocular hypertension treatment (Table 1).

### 4.1. Nitric Oxide-Donating Prostaglandins Analogues

Prostaglandins exhibit a wide range of physiological functions including smooth muscle regulation through interacting with its receptors [5,79]. There are nine PG receptors distributed in both the plasma membrane and nuclear envelope. Among them, FP and EP1–4 were expressed mainly in the uveoscleral tissue in the eye [5,80]. Prostaglandin F2α decreased IOP via FP and EP receptors stimulation, ciliary body (CB) relaxation and pressure-independent outflow increase [11]. Since latanoprost, the first PGF2α analogue, was approved by the FDA for open-angle glaucoma and ocular hypertension treatment in 1996, the use of prostaglandin analogues began to unfold, they offered one of the best IOP lowering effect for glaucoma patients. [11,12]. Prostaglandin analogues reduced IOP by inducing ECM remodeling in the sclera and ciliary muscle. Once FP receptors was activated, stimulation induced metalloproteinase (MMP) enzymes secretion which including MMP1 and MMP9. The MMPs worked by dissolving collagenase and increasing the outflow rate of aqueous humor by the pressure-independent pathway. On the other hand, endogenous prostaglandins synthesis induced by PLA2 stimulation also contributed to the ECM remodeling [5,81,82]. The PG analogues have also been reported to have better control of diurnal IOP compared with the nocturnal IOP, which might be due to the impact from nightly physiological fluctuations on the uveoscleral outflow [83].

In contrast to PG analogues, the modified PGs, NO-donating PGF2α analogues, including latanoprostene bunod (LBN) and NCX 470 showed better IOP lowering effects. They induced both uveoscleral outflow increase by FP receptor stimulation and conventional outflow increase by NO/sGC/cGMP activation [84,86,87]. Vyzulta, a 0.024% latanoprostene bunod eye drop synthesized by Nicox, has been approved by the FDA in patients with POAG and ocular hypertension for IOP reduction [5]. In general, the IOP lowering was achieve in two steps. First, under the action of esterase, LBN was divided into two parts—LA (latanoprost acid) and BDMN (butanediol mononitrate)—the former improved aqueous humour outflow facility via MMP secretion induced by FP receptor activation. Then the butanediol mononitrate was metabolized to NO along with 1,4-butanediol, a byproduct [81,82]. In three animal models, including the ocular hypertensive rabbits induced by hypertonic saline, the glaucomatous ocular hypertensive dogs and the laser-induced ocular hypertensive, BOL-303259-X (also called as PF-3187207 or NCX 116) showed more significant effects in IOP reduction than latanoprost [67].

Clinical trials on the safety, tolerability and IOP lowering effect of latanoprostene Bunod (LBN) have been reported. In the phase 1 study, an open-label KRONUS clinical trial, 24 Japanese adult male subjects were selected according to the required standards of the experiment, and then the baseline of IOP was measured. After 0.024% LBN administration once a night for up to 14 days, the IOP was measured again and the effect of the medication on the subject was evaluated. The results showed that, compared with the baseline value, the IOP of the study group was significantly reduced. In this study, the common adverse reactions were conjunctival hyperemia and punctate keratitis [88]. In the phase 2 study, a dose-ranging study (VOYAGER) was conducted among 413 subjects with ocular hypertension (OHT) or open angle glaucoma (OAG), aiming to find the most suitable concentration of LBN for lowering IOP by comparing different concentrations of LBN and 0.005% latanoprost. Regarding the effect of IOP lowering, the 0.024% dose LBN (decreased by 34 percent) showed significantly better than 0.005% latanoprost did (30 percent). The results demonstrated an additional IOP lowering effect of LBN compared with latanoprost acid (LA) [89]. Another phase 2 trial (CONSTELLATION), among the twenty-four primary open-angle glaucoma and ocular hypertension subjects who participated in the experiment, latanoprostene bunod 0.024% solution showed better night-time IOP lowering effect than timolol maleate 0.5% solution [90]. In phase 3, clinical trials APOLLO and LUNAR were carried out among the subjects with ocular hypertension (OHT) or open angle glaucoma (OAG) in Europe and North America in order to further explore the safety and effectiveness of 0.024% LBN [91,92]. In the studies above, the timolol 0.5% treatment group was regarded as the control group. Both studies showed that after three months of administration, LBN 0.024% exhibited more significant effect in terms of lowering IOP than timolol 0.5%.

NCX 470, a NO donating PGF2α analogue molecule, was synthesized by transforming 15 hydroxyl within bimatoprost into 6-(nitrooxy) hexanoic acid via esterification [84]. In ocular hypertension and glaucoma animal models, NCX 470 showed more effective IOP reduction than bimatoprost. In addition, in both ONT-dogs (ocular normotensive dogs) and OHT-monkeys (ocular hypertensive monkeys), NCX 470 0.042% reduced IOP more than equimolar bimatoprost (0.03%) [84]. However, clinical results of NCX470 is to be released.

### 4.2. Nitric Oxide-Donating Carbonic Anhydrase Inhibitor

The carbonic anhydrase isoforms were expressed in the ciliary body. The isoforms CA I, II, IV and XII, are related to the aqueous humor secretion [93]. Carbonic anhydrase inhibitors (CAIs) were reported to lower IOP by up to 25–30% via inhibiting isozymes in the ciliary body, such as CA II and CA XII [17,18]. Systemic intake of the CAIs, such as acetazolamide, methazolamide and ethoxzolamidelowered IOP significantly. However, due to the ubiquitous distribution of the carbonic anhydrase in the body, systemic side effects, such as metabolic acidosis, weight loss and paresthesia at the extremities have been also frequently reported [94,95,96]. Despite the systemic side effects, CAIs have good application prospects for refractory glaucoma [97]. The good news is that newly developed CAIs have better water solubility and better corneal penetration, such as dorzolamide and brinzolamide, which made it possible to use topical drugs on the ocular surface and have fewer side effects than drugs administered systemically [17,18,96]. However, dorzolamide still has side effects such as depression [98], nephrolithiasis [99], and allergic contact dermatitis (ACD) [100].

Several bi-functional compounds with a NO-donatng moiety bound to a dorzolamide scaffold demonstrated NO mediated effects in the vasculature [19]. Among them NCX 274 and NCX 278 showed ocular hypertensive effects in normotensive rabbits. NCX 274 consisted of a nitrate ester carried by a nitric oxide-donor linker with amino group of dorzolamide through amides, whereas NCX 278 was coupled with carbamates. NCX 274 showed a more powerful IOP lowering effect of NCX 274 than dorzolamide.

### 4.3. Nano-Material Based NO Donors

In contrast to classic NO donors, an increasing number of nano-material based NO donors have emerged aiming to overcome some of the inherent problems of NO donors, such as instability and a short half-life [101,102]. Nanoparticles based NO donors include silica nanoparticles, metal oxide nanoparticles, polymer coated metal nanoparticles and other kinds of nanomaterials (such as dendrimers, micelles) [103]. In additional to IOP lowering [35], it has a wide range of applications including wound healing [104,105], antimicrobial [106,107], cardiovascular diseases treatment [108,109,110,111], erectile dysfunction improvement [112], relieving liver fibrosis [113].

For silica nanoparticles, mesoporous silica nanoparticles (MSNs) and xxx were used as carrier for NO donors [21,22]. Compared with nitroprusside (SNP) solution, the SNP@MSN system exhibited more efficacious and long lasting effect in IOP lowering [21]. With 1/40 dose, SNP@MSN increased IOP lowering effect from 3 hours to 48 hours. For hollow mesoporous organosilica (HOS) nanocapsules, it was biodegradable and can achieve trans-corneal co-delivery of hydrophobic NO donor JS-K (JR) and hydrophilic NO donor L-Arginine (LO) to the target tissues inside the eye [22]. HOS-J_R_L_O_ was endogenous stimuli-responsive and was reduced and oxidized by ascorbic acid and catalysis of eNOS to release a large amount of NO molecules to lower IOP. It successfully treated ocular hypertension in three animal models. HOS-J_R_L_O_ seemed to be a versatile, non-invasive, and efficacious treatment paradigm for precision glaucoma therapy.

Macromolecule composite NO carriers have been also developed to release NO. In β-gal-NONOate-loaded liposomes coated by β-galactosidase-loaded polymer, NO release was accomplished by the catalytic role β-galactosidase played in the catalysis of β-gal-NONOate. Results from C57BL/6 mice showed the conventional outflow facility within theβ-gal-NONOate-loaded liposomes treated group, which, under the effect of β-galactosidase, increased by 84% compared with the vehicle-treated group [23]. This invention gave a good example for NO delivery platform design. Recently, a new polymer combining super cation with GSH (glutathione) responsiveness (PEG-PAspTETA-SNO) was developed as carrier for NO [20]. PEG-PAspTETA-SNO showed more effective corneal penetration and significant IOP lowering effect in both C57BL/6 mice and eNOS knockout mice than the PEGPAspHMDA-SNO control group. Results from AAP cells indicated better uptake for PEGPAspTETA-SNO-RhoB (Rhodamine B labeled polymers) than PEG-PAspHMDA-SNO-RhoB control group. The endocytosis effect of PEG-PAspTETA-SNO-RhoB was inhibited by caveolae inhibitor mβ-CD, which indicated caveolae-Golgi may play a vital role in the PEG-PAspTETA-SNO induced endocytosis.

### 4.4. Other Kinds of NO Donors

The NO release from furoxan can be triggered by mercaptan cofactor. The function of substituents on the ring varied with its arrangement position. The third group of substituents is in charge of the total amount release of NO, and the fourth one is responsible for the balance of hydrophilic-lipophilic [85]. Blangetti et al. developed a big array of furoxan derivatives based on the basis above, the IOP lowering effects of which were tested in a transient ocular hypertensive rabbit model (tOHT). Some compounds showed similar IOP lowering effect to timolol after an hour of dosing. The study concluded that it was not the amount of NO released but the hydrophilic-lipophilic balance that determined the IOP lowering effects of such kinds of furoxan derivatives.

## 5. Challenges Associated with NO Donors

Although NO donor Vyzulta has been approved by the FDA for the treatment of glaucoma and ocular hypertension, some problems remain to be solved. Nitrate tolerance or organic nitrates’ invalidation occurred among coupled NO donors during continuous therapy [114,115,116]. The formation of s-nitrosothiols and nitration is the product of the reaction between NO and protein [117], and it is also a potential factor that causes damage to the conventional outflow pathway and ocular hypertension [118]. A recent study showed that a single dose of SNP@MSNs (NO donor) in wild-type mouse eyes can lower the IOP (up to 48 h), but continuous use of SNP@MSNs caused an increasing IOP (from the sixth day on). However, co-application of SNP@MSNs with MnTMPyP (antioxidant) could not only prevent IOP increase, but also increase the magnitude of IOP lowering [118]. This experiment revealed the problem of peroxynitrite (ONOO-) accumulation caused by continuous release of NO. Further research in this direction will help avoid side effects and improve the efficacy of NO donors.

## 6. Conclusions

NO donors have made great progress in the treatment of various diseases, especially in glaucoma and ocular hypertension. Future development of NO donors should focus on effective topical delivery (as eye drops), longer duration of action and minimizing long term side effects. In order to achieve these goals, it is important to continue exploring in designing and synthesizing better NO donors. New attempts will help to develop better and more treatment option for the well-being of glaucoma patients.

## Figures and Tables

**Table 1:** Classifications of NO-donating drugs.

NO-Donating Drugs		Underlying Mechanisms Involving IOP Lowering
NO-donating Prostaglandins	LBN [81,82]	ECM digestion, sGC stimulation TM relaxation
	NCX 470 [84]	PGF2a and NO/cGMP pathways activation
NO donating CAIs	NCX 274, NCX 278 [19]	carbonic anhydrase type-II isozyme inhibition, NO/sGC/cGMP pathway stimulation
Nano-materials based NO donors	PEG-PAspTETA-SNO [20] SNP@MSNs delivery [21]	concentrated endogenous GSH-triggered NO release NO-cGMP pathway stimulation
Other kinds of NO donors	Furoxan derivatives [85] β-gal-NONOate [23]	NO-cGMP pathway stimulation NO release via β-gal-NONOate enzymatic biocatalysis

NO, nitric oxide; IOP, intraocular pressure; LBN, latanoprostene bunod; ECM, extracellular matrix; sGC, soluble guanylate cyclase; NCX 470, NO-Donating bimatoprost; NCX 274, NO-Donating CAI compound 4; NCX 278, NO-Donating CAI compound 6.

## Abbreviations

NO, nitric oxide; IOP, intraocular pressure; LBN, latanoprostene bunod; ECM, extracellular matrix; sGC, soluble guanylate cyclase.
